# Precise Therapy Using the Selective Endogenous Encapsidation for Cellular Delivery Vector System

**DOI:** 10.3390/pharmaceutics16020292

**Published:** 2024-02-19

**Authors:** Vacis Tatarūnas, Ieva Čiapienė, Agnė Giedraitienė

**Affiliations:** 1 Institute of Cardiology, Lithuanian University of Health Sciences, Sukileliu 15, LT 50103 Kaunas, Lithuania; vacis.tatarunas@lsmu.lt (V.T.); ieva.ciapiene@lsmu.lt (I.Č.); 2 Institute of Microbiology and Virology, Lithuanian University of Health Sciences, Eiveniu 4, LT 50161 Kaunas, Lithuania

**Keywords:** retroviruses, virus-like particles, drug delivery, precise therapy

## Abstract

Interindividual variability in drug response is a major problem in the prescription of pharmacological treatments. The therapeutic effect of drugs can be influenced by human genes. Pharmacogenomic guidelines for individualization of treatment have been validated and used for conventional dosage forms. However, drugs can often target non-specific areas and produce both desired and undesired pharmacological effects. The use of nanoparticles, liposomes, or other available forms for drug formulation could help to overcome the latter problem. Virus-like particles based on retroviruses could be a potential envelope for safe and efficient drug formulations. Human endogenous retroviruses would make it possible to overcome the host immune response and deliver drugs to the desired target. PEG10 is a promising candidate that can bind to mRNA because it is secreted like an enveloped virus-like extracellular vesicle. *PEG10* is a retrotransposon-derived gene that has been domesticated. Therefore, formulations with *PEG10* may have a lower immunogenicity. The use of existing knowledge can lead to the development of suitable drug formulations for the precise treatment of individual diseases.

## 1. Introduction

Advances in the understanding of molecular disease mechanisms, biochemical processes, and molecular pharmacology have led to the understandable need to target specific cells or tissues and signaling pathways involved in disease progression. In line with this, advances in research and technology of drug development are paving the way to target specific cells or tissues and signaling pathways involved in disease progression and to develop tailored, advanced, and innovative drug formulations that can affect the specific site. High drug concentrations at the target site or receptor are achieved, side effects are minimized, and therapeutic efficacy in treating disease is dramatically increased. This precise therapy has advantages over conventional drug formulations, e.g., tablets or capsules [1]. Conventional dosage forms are associated with a number of issues such as low bioavailability, frequent use, side effects, or lack of patient compliance [2]. The use of nanoparticles, liposomes, or other available forms for formulations of drugs could help to overcome these issues. Nanoparticles could be engineered to encapsulate and deliver drugs to specific targets. The use of current nanotechnologies has enabled the development of nanoparticles that can release drugs in response to specific stimuli such as light, magnetism, or pH changes [3]. Precision medicine and pharmacogenomics will undoubtedly play a role in the discovery of tissue-, organ- or cell-specific drug formulations and in the development of customized therapies. Therapies will be tailored to minimize adverse drug effects but maximize therapeutic activity in individual patients according to their genetic information, family history, lifestyle, metabolic data, etc. [4].

### Virus-like Particles (VLPs) as a Strategy for Targeting Specific Tissues and Cells

Drugs can often target non-specific areas and cause both desired and undesired pharmacological effects. This makes the discovery of new molecules a very complicated, costly, and unpredictable process [5]. For example, cytotoxic drugs usually do not distinguish between normal (blood marrow cells, skin, scalp, and stomach cells) and cancer cells that grow quickly. Thus, drugs have a toxic effect on healthy cells [6]. A large number of drugs are converted by hepatic enzymes into active compounds that can act on target receptors. Most of them are metabolized by the cytochromes P450 [7]. Mammals produce cytochromes P450 (CYP450) in various cells, tissues, and organs (liver, kidney, brain, heart, adrenal gland, skin, etc.). The cytochromes are membrane-bound enzymes with some exceptions [8]. Normally, the expression of CYP450s is localized in certain cells and associated with certain physiological functions of tissues and organs or cells, or they may be localized in different subcellular compartments. The endoplasmic reticulum and the mitochondria are the places where CYP450s can be found most frequently. CYP450s can also be detected at the outer nuclear membrane, various Golgi compartments, peroxisomes, and plasma membranes [8]. Targeting diseased cells could be a challenge. Healthy cells may have higher CYP450 activity, which means that drugs that target fast-growing diseased cells (such as cancer cells) may have an even higher effect on normal cells as well [9].

Direct delivery of the target-specific therapy by using virus-like particles (VLPs) is a promising strategy for precise therapy. There are various strategies for making treatment more efficient and avoiding adverse effects of drug therapy. The introduction of genes coding for drug-activating enzymes—GDEPT (Gene-Directed Enzyme Pro-Drug Therapy), the introduction of transgenes using viruses (vectors), called VDEPT (virus-directed enzyme prodrug therapy), and ADEPT (antibody-directed enzyme prodrug therapy), in which antibodies conjugated with an enzyme are used against target cells [10,11].

## 2. Viral Nanoparticles

Viral nanoparticles (VNPs) or non-infectious virus-like particles (VLPs) are being explored as self-assembling, efficient, and adaptable drug delivery systems used in various applications [12,13]. VNPs are nanomaterials that are derived from viruses (human, bacterial, plant). VLPs are genome-free structures of viruses [14]. VNPs are used for the encapsulation of drugs and their delivery through biological barriers to the target site where they are needed. However, host immunogenicity may remain an obstacle to the clinical use of certain VNPs. Recognition of viral components can trigger an immune response that leads to clearance of them from the organism [13].

Virosomes are a kind of nanoparticles. They were first produced after the inclusion of viral spike proteins in a liposome [15]. In 2018, Donaldson and colleagues defined enveloped VLPs as nanoparticles that lack the capsid protein [16]. Virosomes are usually obtained from viruses. Influenza viruses could be the most common source among them, due to the specific characteristics of interaction with human tissues and the immune system. It is known that these virosomes can induce human immune activity [17,18]. Therefore, the use of various viral vectors may be very practical for the development of drug delivery systems (formulations). Viral nanomaterials surpass the properties of synthetic nanoparticles used for medical applications. They possess an ability to cross biological barriers, interact with target cells and receptors, and evade the immune system more efficiently than synthetic biomaterials. However, to date, there is still no significant improvement in biomedical nanomaterials or formulations which are based on viral traits. Synthetic nanoparticles, compared to viral nanoparticles, are safer and more flexible for use [19].

## 3. Production of VLPs

Different platforms can be used for the production of VLPs, such as yeast [20] bacteria, insects, plants, mammalian cells, or even cell-free expression systems. The quaternary viral capsid proteins can be individualized according to the parameters of the expression system [21,22]. Chimeric VLPs can contain structural proteins from different viruses [23].

The most important steps in the production of VLPs are production (upstream processing), purification (downstream processing), and formulation [24,25,26]. The concept is based on the production of a clone of viral structural proteins and the expression of these proteins in self-assembling format in expression systems (bacteria, plants, mammalian cells). Cultivation and lysis (or mechanical disruption) of the cells is followed by clarification (filtration or precipitation), purification (main step, usually chromatography is used), and polishing of the target VLPs [27,28].

Bacterial cell cultures (such as *Escherichia coli*, *E. coli*) were investigated as a platform for VLP production [29,30]. Bacterial and yeast systems, however, may produce VLPs that can be contaminated with residual elements of the host cells such as nucleic acids, lipids, or proteins. These contaminants may stimulate a response in the human organism when treated [31]. The most suitable environment for correct VLP assembly is mammalian cell cultures. Mammalian cells are used to produce large proteins, but the introduction of the desired gene could be expensive and time consuming [32,33]. CHO (Chinese hamster ovary cells) are preferred over human cells for the production of VLPs because they are not susceptible to human viral infections, but high contamination with fetal albumin could be another disadvantage [34,35]. Plant-based environments are suitable for the production of a large numbers of VLPs and cheap [36] and may enable one to avoid infection [37].

## 4. Bacteria and Their Vesicles

Bacterial membrane-derived vesicles (BMVs), traditionally considered as microbial metabolic wastes, are extracellular substances spontaneously released by bacteria in culture. If bacteria are activated or stimulated, the production of BMVs is increased [38]. BMVs are spherical, bilayered proteolipids that transport bacterial virulence factors, bioactive proteins, lipids, and nucleic acids [39]. The role of BMVs is discussed very intensively as they facilitate intracellular communication, may alter a composition of the microbiota, take part in the formation of biofilms, assist in the uptake of nutrients, and serve as a chemical waste removal system for bacteria. Moreover, BMVs are a tool for bacteria to interact with host; they may overwhelm the host immune system or injure host tissues [40].

Bacterial membrane-derived vesicles mainly are secreted from the outer or cell membranes [41]. Depending on bacteria’s differentiation by Gram staining, vesicles are divided into two large groups: Gram-positive extracellular vesicles and Gram-negative extracellular vesicles and depending on distinct categories: outer-membrane vesicles (OMVs), outer–inner membrane vesicles (O-IMVs), cytoplasmic membrane vesicles (CMVs), and tube-shaped membranous structures (TSMSs) [42]. Gram-negative bacteria-derived OMVs are composed of inner (phospholipid) and outer (lipopolysaccharide (LPS), outer membrane proteins, and periplasmic proteins) leaflets and occasionally DNA or even RNA [43]. Additionally, to all the functions of extracellular functions, OMVs also act as decoys for immune factors, antimicrobial compounds, and bacteriophages [38]. O-IMVs are double-bilayer vesicles described as budding/blebbing cells of *Shewanella vesiculosa* that mediate the transfer of local intracellular components [44]. Only recently, it was confirmed that Gram-positive bacteria also release extracellular vesicles called CMVs. The composition of the cytoplasmic membrane and CMVs is very similar’ it is thought that CMVs undergo changes within the cytoplasmic membrane [45]. CMVs carry various cargo compounds such as nucleic acids, viral particles, enzymes, and effector proteins that play parts in bacterial competition and survival (e.g., *Staphylococcus aureus* CMVs are enriched in virulence proteins including superantigens, hemolysins, Staphopain A, coagulation factors, IgG-binding protein SbI, lipase, *β*-lactamase, and N-acetylmuramoyl-l-alanine amidase [41], and they are involved in material exchange, host immune evasion, and modulation [46]. TSMSs are produced by Gram-positive and Gram-negative bacteria and known as nanotubes, nanowires, or nanopods. Their role is to connect bacteria for material exchange and in biofilms at the periplasmic level for social activities [47].

## 5. Bacterial Extracellular Vesicles as Bioactive Nanocarriers for Drug Delivery

Bacteria-derived extracellular vesicles (or nanovesicles) are a promising tool for novel therapeutic drug delivery systems [48]. The most used bacteria in preclinical and clinical treatment trials are *Streptococcus*, *Clostridium*, *Bifidobacterium*, *Listeria*, *Escherichia*, *Lactobacillus*, and *Salmonella* [49]. They are the most commonly used expression system for the production of recombinant proteins and VLPs. Bacteria do not have a system for post-translational modifications (PTM). Furthermore, they cannot form a complete disulfide bond, have problems with protein solubility, and cannot be used for enveloped VLPs [50]. Bacteria-based VLPs are usually inexpensive and robust. Thus, it makes these expression systems widely used. *E. coli* is among the most common commercial bacterial cell cultures for VLP production [51]. The lack of PTM control of the proteins, but the low production price and rapid growth of *E. coli* cell cultures, makes them suitable to produce small protein molecules and generate chimeric VLPs [51] with a higher level of impurities compared to other expression systems (such as yeasts) [31,52]. Recently, Tamburini et al. demonstrated that bacterial OMVs could be an efficient and inexpensive solution for the production of anti-HPV vaccines. Conserved viral L2 epitopes can be expressed on the surface of OMVs. This study was performed using the outer membrane system of *E. coli* [53].

## 6. Yeast and Their Use for the Production of VLPs

Yeast cell cultures are used for the production of recombinant proteins and VLPs. Yeast, in particular *Saccharomyces cerevisiae* and *Komagataella phafii* (formerly *Pichia pastoris*), are well recognized systems for the expression of VLPs. They fall under the group of generally recognized as safe (GARS) cell cultures [54,55]. Both *S. cerevisiae* and *K. phafii* expression systems are robust, inexpensive, easy to genetically manipulate, and, unlike bacteria, free of endotoxins [52]. Cultivation of *K. phafii* on a commercial scale shows rapid growth and a very concentrated cell density [56]. Yeasts are versatile platforms as they can express different forms of products (antigens). Yeasts have complete genome sequences that are well studied. However, one of the main advantages of yeasts is that they can secrete biotechnologically produced proteins into the culture medium. This facilitates the extraction and purification of proteins of interest [57]. Compared to *S. cerevisiae*, *K. phafii* has a shorter and less immunogenic glycosylation pattern and a higher protein yield due to the higher density of cell cultures [58]. *Firdaus* et al. performed HPV52 L1 protein expression analysis in *Pichia pastoris* and *Hansenula polymorpha* yeasts. Researchers showed that self-assembled VLP formation and stable integration during protein induction were successful in both yeasts. These expression systems are used for vaccine production in the industry; however, the production effectiveness of desired proteins differs among yeasts [59].

Products that use non-humanized glycans are only suitable for human consumption to a limited extent. Protein glycosylation differs in humans and yeasts [60]. Yeast is not able to produce humanized glycans. This places considerable demand on their physicochemical parameters, their stability, and their immunogenicity [61]. Studies on humanized glycosylated end products in yeast are under development. There are also a number of yeasts that have never been studied for the production of humanized glycans [62]. Moreover, yeasts cannot produce enveloped VLPs [63]. However, enveloped VLPs are poorly characterized due to their structural non-uniformity compared to non-enveloped VLPs [64].

## 7. Mammalian Cells for VLPs Expression

These expression systems allow one to make both enveloped and non-enveloped VLPs. They have the most precise PTMs as compared to other expression systems. Large and complex proteins can be produced by these cells. However, introduction of a gene is time-consuming if we compare it to other expression systems [32,33]

Human embryonic kidney 293 (HEK293), human amniocytes CAP-T, Vero-9, ELL-o, as well as CHO (Chinese hamster ovary cells), and baby hamster kidney-21 (BHK-21) are used for the production of VLPs [22]. CHO cells are preferred over human cells because they are not susceptible to human viral infections. In addition, CHO cells have several advantages that make them suitable for expression systems. The post-translational modifications in these cells are comparable to those of human cells. They do not produce immune galactose–galactose epitopes. In addition, the proteins produced by CHO cells are secreted into the cell culture medium. The cells can be grown in a suspended or adherent state. The main disadvantage of CHO cells is that contamination may be higher due to the unfavorable use of 10% fetal bovine albumin [34,35]. Other disadvantages include the low yield of target proteins, expensive production, long time to produce a protein, and infection with mammalian viruses [65].

## 8. Molecular Farming or Plant-Based VLPs Production

Current manufacturing technologies using mammalian cells cannot meet the annual demand for biopharmaceutical products. However, plants can produce large quantities of products. The production process of biopharmaceuticals by plants is known as molecular farming. When plants are used, this process can be relatively safe and cheap, and it can also easily scale up production [36]. Plant-based viruses do not infect humans. This ensures the safety of plant-based bio-pharmaceuticals [37]. Viral vectors, such as potato virus X, tobacco mosaic virus, and cowpea mosaic virus, have been used for the rapid expression of recombinant proteins. Nowadays, industrial processes complying with good manufacturing practices are performed using plant-based manufacturing [66]. However, there is another issue to overcome. Sialylation is responsible for glycoprotein durability. Therefore, the degree of sialylation is important because it can affect the function of the VLP. Izadi et al. showed that there are different methods for making plants produce human-like protein sialylation. A multigene vector can be used to simplify these processes [67]. Plants make it possible to make the desired assembly of VLPs without contamination of the product with human or animal proteins. Therefore, it could become a favorable expression system for large numbers of VLPs.

A wide range of expression systems, including prokaryotic cells, yeast, plants, and mammalian cell lines, can be used to generate VLPs. The main advantages and disadvantages are presented in Table 1. However, cell-free expression systems may also be of interest. VLP proteins are produced by cell-based systems and then folded correctly in a cell-free environment. The main disadvantage is that these expression systems are expensive. [21].

## 9. What Are Retroviruses and Human Endogenous Retroviruses?

Retroviruses are encapsulated reverse-transcribing viruses with single-stranded RNA (ssRNA) genomes. These RNA viruses have the ability to produce a double-stranded DNA copy of their genome during their replication cycle in the host cell upon infection. Retroviruses, belpaoviruses, metaviruses, and pseudoviruses share capsid and nucleocapsid proteins [68,69]. According to the complexity of their genomes, retroviruses might be classified into two groups: alpha, beta, gamma, and epsilon viruses have simple genomes, while lentiviruses, deltaviruses, and spumaviruses have more complex genomes [70].

Retroviruses play a crucial role in human evolution and divergence of the hominids [71]. Vertebrate genomes have integrated sequences of human endogenous retroviruses (HERVs), which were acquired during the evolution of vertebrate organisms [72]. In humans, retroviruses make up more than 8% of the human genome [73,74], and nowadays they are more likely symbionts than parasites [75]. As an example, two HERVs encode the functional proteins syncytin-1 and 2 which are very important during the embryogenesis of fetuses. These proteins participate in placenta formation and the adaptation of the maternal immune system to the fetus [73].

Endogenous retroviruses (ERVs) were first identified by combining immunological, virological, and Mendelian genetic methods in the late 1960′s and early 1970′s [70]. Now, it is well known that ERVs and LTR (long terminal repeat) retrotransposons make up a substantial part of the human genome. These sequences were integrated into the human genome millions of years ago [73,74].

ERVs are transcriptionally silenced, but with aging, abnormal activation of retrotransposons is observed. It is explained by the loss of heterochromatin during aging [76,77]. Autoimmune and aging diseases related to HERV activation are induced [78].

HERV-K is the most recent virus in modern humans. It is thought that the virus entered the human genome nearly 1 million years ago, whereas ERV-L existed over 70 million years ago. ERV-W is shared by multiple animal and plant species, which allows us to hypothesize that this virus began endogenization a long time ago [79]. HERV-K is the sole human-specific member of ERVs, which sometimes might have expression of functional proteins in certain tissues and cells. HERV-K constitutes approximately 5% of human transposable element insertions. The expression of these ERV proteins is upregulated in breast, ovarian, melanoma, and lymphoma cancers or other diseases such as rheumatoid arthritis [80].

The first discovery of retroviruses as tumor-causing viruses was more than 100 years ago [81]. In 1981, immunosuppressed patients were detected [82,83,84], and the virus which caused the immunosuppression was isolated 2 years later in 1983 by researchers from the Institut Pasteur [85].

**Figure 1:**
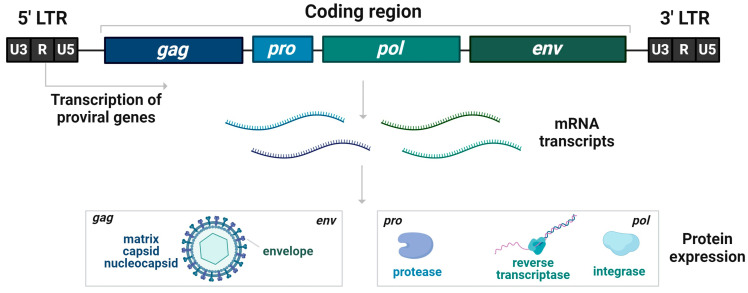
Genomic structure of full-length protein-coding HERVs. The coding region comprising *gag*, *pro*, *pol*, and *env* is flanked by two long terminal repeats (LTRs) that provide regulatory elements such as enhancers, promoters, and polyadenylation sites. Complete genomic structure HERVs are less common than nonprotein-coding HERVs because of the accumulation of mutations in their DNA sequence.

The classical genomic structure of HERVs (Figure 1) is made of an internal region of four genes, *gag*, *pro*, *pol*, and *env*, flanked by two inverted repeats of non-coding regions comprising regulatory functions (LTRs). The capsid, nucleocapsid, and matrix proteins are encoded by *gag*, protease by *pro*, and transcriptase and integrase by *pol* when the envelope protein is encoded by *env* genes. HERV DNA sequences are often truncated, with mutations, insertions, and deletions, but also contain complete copies [86].

HERV regulates the initiation and progression of different types of cancer [87]. Due to demethylation of the HERV-K sequence, an increased expression of GAG protein in teratocarcinomas was determined [88] when increased GAG mRNA expression is associated with poor prognosis in breast cancer [89]. Reis and colleagues reported that prostate cancer progression correlates with increased humoral response to a human endogenous retrovirus GAG protein. Anti-HERV-K *Gag* antibody titer is higher in stages III and IV of cancer compared to stage I and II, which predicts worse survival [87].

Other viral proteins encoded by the *pro* gene might be candidates in causing diseases. It was suggested that cellular proteins are potential substrates of the HERVs protease (Pro). Expression of Pro has an impact on cell biology, and it may be related to human diseases [90].

*Pol* gene encodes polyproteins which are then processed into individual proteins [91]. Bergallo et al., 2017 found relative overexpression of the HERV-K *Pol* mRNA molecule in bone marrow cells from the majority of analyzed lymphoblastic leukemia samples [92].

The fourth gene of the classical structure of HERVs is the *env* gene, which encodes a viral envelope that is needed to recognize a receptor and for the process of membrane fusion. Additionally, the *env* gene is important in the classification of HERVs from other LTR retrotransposons. It is known that the env protein induces cell–cell fusion and epithelial mesenchymal transition (EMT), which could contribute to tumorigenesis [93]. The env protein can play a role in tumorigenesis and metastasis of breast cancer as its expression of artificial regulation can impact the expression of tumor-associated genes, cell proliferation, migration, and invasion [94].

## 10. Drug Delivery of the Future: “Humanized” Virus-like Particles

Local administration is a basic drug delivery method, but it is limited by physiological barriers to tissues, cells, and organs (such as the mucus barrier) [95]. Innovative ways of delivering molecules to target organs or cells are needed to overcome these barriers. The use of nanoparticles for molecule delivery remains challenging due to nanoparticle accumulation in the liver and clearance by the reticuloendothelial system [96]. Chemical conjugation in drug delivery systems, e.g., a target drug with small chemical molecules, has enabled the preparation of clinically active and effective antibody–drug conjugates [97]. The use of hydrophobic moieties has improved the transport of DNA/RNA oligonucleotides through the blood–brain barrier in rodents [98].

Biological vectors, nano- or microsized particles from the cells, usually consist of structural components of the cell membrane, extracellular vesicles, and exogenous carriers of genetic information, such as viruses [99]. The latter, viral vectors (viral nanoparticles), are natural nanomaterials derived from plants, mammals, and bacteriophages. Viral nanoparticles are genome-free versions and can encapsulate various substances to target, for example, tissue-specific shells. Their main advantages are biocompatibility and biodegradability. They can be used as native substances, allowing us to overcome undesired clearance from the organism [14]. Viral nanoparticles are unique and easily modified. The interiors of these particles can be used to encapsulate and protect various substances, while the exteriors can be adapted to bind specific molecules to different targets [100]. Today several lentiviral- or retroviral-like particles are being developed for the delivery of RNA, proteins, and ribonucleoproteins [101,102,103,104,105,106,107]. Retroviruses can encapsulate both their own and the host’s RNA [108].

Mass-spectrometry (MS)-based analysis of different mammalian cells has shown that mRNA and protein abundance varies between cells. Genes coordinate patterns at the transcriptomic and proteomic levels [109]. Viruses incorporated into the human genome may be considered for use in delivery systems for therapeutic molecules, such as RNA.

**Figure 2:**
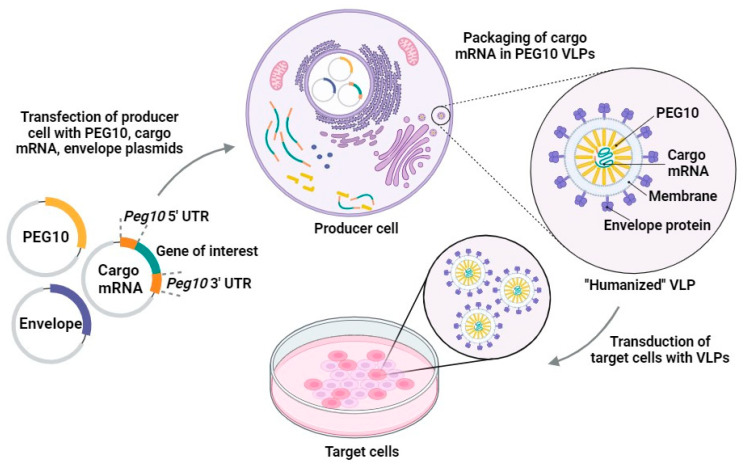
Scheme representing the SEND system discovered by Segel and colleagues [103]. The main focus of this system is the functional delivery of the desired cargo RNA flanked by *PEG10* UTR sequences. Producer cells transfected with engineered cargo mRNAs result in the packaging and export of PEG10 VLPs. Translation of “humanized” VLPs is verified in recipient cells. Reprinted with permission from the AAAS.

Endogenous retroviruses (retrogenic viruses) have lost their original functions, but they may play important roles in human physiology, as their genes may produce specific proteins [73]. Retroelements retain their functions and play a role in the binding and transfer of mRNA and the formation of capsids in the cell [110]. Arc, which is a *gag* homolog, regulates inflammatory processes in the skin [111]. *PEG10/MART2* is observed during embryonic development [112,113]. Researchers from a group of pioneer developers of CRISPR-Cas9 technology proposed the use of a novel model of vectorization based on virus-like particles produced by retroelements of the human genome [103]. Segel et al., reported several *gag* homologs of the capsid protein, which forms VLPs and an LTR retrotransposon homolog, *PEG10* (paternally expressed gene 10), which is involved in vesicular expression of its own messenger RNA [103]. Researchers have shown that the human PEG10 protein can form VLPs and transport its own mRNA. Other mRNAs flanked by the *PEG10* gene might also be encapsulated. Based on these data, the authors described the first endogenous system for an RNA delivery system based on HERV: a “selective endogenous encapsidation for cellular delivery” (SEND) system (Figure 2). This system can use endogenous proteins for the production of VLPs [103].

## 11. What Is *PEG10*?

Paternally expressed gene 10 (*PEG10)* is a retrotransposon-derived gene necessary for development in mammals. It is a paternally expressed imprinted gene derived from the Ty3/Gypsy LTR retrotransposon lineage known as Sushi-ichi. *PEG10* codes for two domains: the *gag* and *pol* domains. These two domains are separated by a programmed ribosome frameshifting site [114,115]. During ribosomal frameshift, two proteins are translated: *gag* and *gag-pol* (*PEG10-RF1* and *PEG10-RF1/2*). These proteins may interact with the TGF-β receptor family and initiate the beginning of disease [116]. PEG10 activity was found to affect cancer growth [117,118,119]. Studies also showed that PEG10 stabilizes RNA during typical placental development [114]. A study showed that a mouse that was knocked-out for *PEG10* showed early embryonic lethality due to placental defects [120].

The PEG10 *gag-pol* protein cuts itself and generates a free nucleocapsid fragment which adjusts gene expression in the nucleus [121]. UBQLN2 (proteasome shuttle factor ubiquilin 2) controls *PEG10* expression by breaking it down [121,122]. The human *RTL8* gene restrains *PEG10* by incorporating it into VLPs [123]. Another study found that PEG10 may impact cell viability and prevent apoptosis [124].

VLPs can be built from endogenous human proteins which are based on PEG10. This approach has an advantage of reducing immunostimulation and the toxicity of mRNA-containing VLPs transporting into the cytoplasm [125]. Certain human retroelements retain some of their functionality. They bind and transport mRNA within the cell [110].

Let us say we have a drug carrier of the future: a “Humanized” Virus-Like Particle. The following problems remain to be solved: 1. designing a drug that has a molecule that matches the target we want to target, and 2. tailoring the specific carrier to properly carry the drug to where it is needed. Genomics is key to resolving both problems.

## 12. Pharmacogenomics

**Figure 3:**
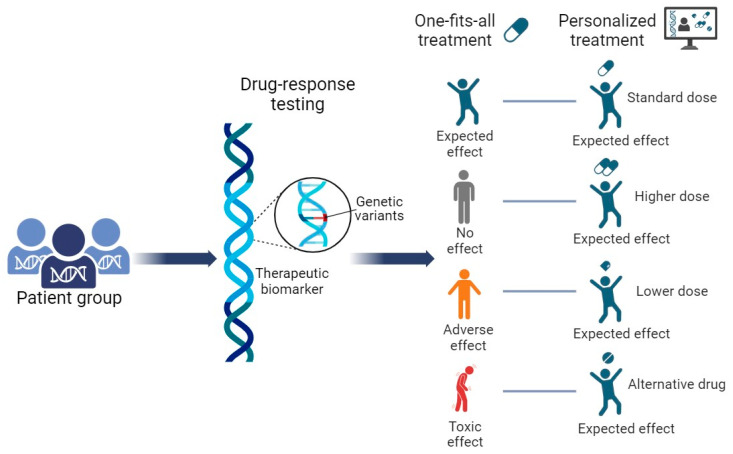
The concept of pharmacogenetics/pharmacogenomics: the right drug and the right dosage for the right patient. This picture illustrates the concepts of gene-based drug dosing. In the past, classical clinical algorithms recommended the same drug or the same drug dosage for everyone. Observations have shown that the drug can produce different effects in different patients. These may be a normal (expected) therapeutic effect, no therapeutic effect, or adverse effects or even a toxic effect, depending on the genotype of the patient. Therefore, pharmacogenomic-based algorithms nowadays recommend adjusting the drug dosage to the patient’s genotype.

Interindividual variability in drug response is a huge problem when pharmacological treatment is prescribed. A person may have individual differences that may be acquired during his life or inherited from parents through genetic material. In 1983, already 40 years ago, Sweeney wrote that variations in drug response may be pharmacodynamic or pharmacokinetic [126]. The pharmacodynamic response indicates the different strength of the pharmacological effect on the receptor at the same drug dose. Pharmacokinetic means that a person can have a different concentration of the active substance molecule at the same drug dosage. Nowadays, it is obvious that the pharmacological profile of each individual person is encoded by the genes (Figure 3). Pharmacogenomics combines pharmacology and genomics to select safe and effective drugs, formulations, and dosages for individuals [127].

The observation that patients react in different ways when exposed to the same active substance gave rise to the idea of pharmacogenetics/pharmacogenomics (PGx). The first documented recognition of side effects in some individuals was described by Pythagoras around 510 BC. In 1932, Snyder described an autosomal recessive trait of the phenylthiourea non-taster phenotype [128]. In 1959, Vogel was the first to describe a modern concept according to which the effect of drugs can be influenced by human genes. He used the term “pharmacogenetics” to describe this effect [129]. Motulsky, one of the founders of tailored drug therapy, stated that “any drug response that is observed more frequently in a particular racial group when other environmental variables are the same usually has a genetic basis” [130].

The first pharmacogenetic studies were not able to explain different responses to drugs. Nowadays, improvements in molecular biology tools and technologies has led to a deeper understanding of processes in cells, organs, and organisms [131]. Lower prices per analysis has made it possible to discover more new variants in the genomes of individuals and to predict the results of drug treatment more precisely. The possibility of using advanced methodologies with high accuracy in predicting the effect of certain compounds has transformed pharmacogenetics to pharmacogenomics. The first sequencing analysis of the human genome has paved the way to a deeper understanding of how genes can influence drug action. In this way, reference genomes have fueled technological progress in genomics [74,132].

To date, guidelines for treatment individualization have been validated [133]. Variants in genes involved in the absorption, distribution, metabolism, and excretion of drugs (ADME) have been included in these guidelines. Based on these guidelines, a precise or individualized treatment has been planned according to the genotype of each patient in order to achieve the right drug and right drug dosage for each patient. Due to challenges in translating PGx data into clinical practice, such as changes in current clinical protocols, and, most importantly, rare pharmacogenomic variations with unclear functional consequences, there still remains a knowledge gap regarding PGx [134,135].

Drug manufacturers recommend the genotyping of certain gene variants on their package inserts in order to predict the “right drug dosage” and the “right drug” for the patient. However, there is no consensus between the organizations that approve the use of drugs (e.g., European Medicines Agency (EMA), Food and Drug Administration (FDA)) and the working groups of pharmacogenetics/genomics experts such as the Clinical Pharmacogenetics Implementation Consortium (CPIC) and the Dutch Pharmacogenetics Working Group (DPWG) [136]. According to Lauschke, only 22% of FDA designations refer to pharmacokinetic biomarkers and 66% to mutations in the somatic genome of tumors. In contrast, 52% of EMA labelling refers to pharmacokinetic data. The labels of the EMA and the expert organizations (CPIC, PGWP) overlap by 9%, while the FDA reaches 40% [136].

Nowadays, clopidogrel can be prescribed based on pharmacogenomic knowledge. Based on *CYP2C19* gene variants, optimal antiplatelet therapy can be recommended before the first dose. It is recommended that patients with intermediate or poor CYP2C19 metabolizer status avoid clopidogrel. Alternative therapy with ticagrelor and prasugrel may be the treatment of choice [137]. Warfarin is another drug that also receives attention. Warfarin is a blood-thinning drug (anticoagulant). It is prescribed to patients at high risk of venous thromboembolism. Patients using warfarin may experience bleeding or thrombosis events due to high dosage–effect variability between patients. Studies have shown that *CYP2C9*, *VKORC1*, and *CYP4F2* gene variants significantly affect the correct dosage of warfarin. In addition, patient age, concomitant use of drugs, and clinical state may determine the required dosage of warfarin for each individual. [138].

## 13. Pharmacogenomics Approaches: New Drugs for Epigenome Control

Investigations from various PGx studies have shown the heterogeneity of the results. Large scale, genome-wide studies have allowed us to make discoveries in pharmacogenomics [139]. Discoveries are usually made with two approaches: reverse and forward.

Reverse genetics is used to determine which phenotype can be produced when the identity of the gene is known. The phenotypic effects can be studied in different tissues, environments, etc. A clustered regularly interspaced short palindromic repeat (CRISPR)-based technology has revolutionized genetic engineering [140,141]. Since its discovery in 1987 [142], this technology has greatly improved. Nowadays CRISPR-Cas are represented by two classes and six types and numerous subtypes [143]. CRISPR-based genetic engineering enables experiments with ADME enzymes, which are crucial for understanding the pharmacogenomic profile (phenotype effect) and for treatment individualization at the DNA and RNA level [144,145]. CRISPR technology is also employed in forward genetics. Forward genetics is used to investigate a genetically heterogeneous population in a controlled setting in order to uncover genetic modifiers that influence drug activity. Forward genetics identifies the genetic basis of specific phenotypes [146].

Advances in new molecular biology techniques such as next generation sequencing (NGS) have shed light on variations in the genome, such as structural variations (SVs), which have been less studied so far. SVs are genome variations of 50 bp in size. SVs may have more severe effects because of their size compared with single-nucleotide polymorphisms [147]. SVs affect about 3.4 times more nucleotides in human genomes and may have a substantial impact on PGx and drug dosage [148,149]. Rare variants can contribute to between 10 and 40% of genetically encoded functional variability [150].

Several studies have reported that epigenetic modifications of genes can influence the pharmacodynamics and ADME of drugs [151,152,153]. Different ADME enzymes (CYP1A2, CYP1B1, CYP2C9, CYP2C19, CYP2D6, and CYP3A4) change their expression level depending on their promoter methylation [153]. During liver maturation, CYP3A4 and CYP3A7 are differentially expressed. CYP3A4 is expressed in adults, whereas CYP3A7 is expressed in the fetal and neonatal stages. Researchers have shown that CYP3A4 is induced by PXR-mediated activation during rifampin treatment, leading to changes in the histone profile [154,155]. In addition, epigenetic/genomic profiles are specific to cells and tissues or diseases [156,157]. Epigenetic-targeting drugs, or epidrugs (azacitidine, decitabine, vorinostat, romidepsin, belinostat, panobinostat, enasidenib, ivosidenib, tazemetostat), are nowadays approved by regulatory agencies for clinical use [158,159].

Developing technologies and methods generates large amounts of data. This poses problems for researchers working in this field. As described above, various factors can affect gene activity in cells, tissues, or organisms. Therefore, additional effects of these variations can be observed. Rare variants discovered by new, more powerful methods will generate more data. These rare variants may have synergistic or antagonistic effects. There will also be interactions between genes, drugs, and the environment. Therefore, precision medicine will undoubtedly include models for drug dosage prediction and treatment individualization based on large data sets that consider the complexity of gene variants carried by the patient, gene expression, epigenomics, environment, etc. [160].

## 14. Pharmacogenomics—A Tool for Precise Development of VLPs: Vaccinomics

VLPs are a promising tool for future drug formulations and precision medicine. They are desirable, safe, and biocompatible delivery vehicles for drug-delivery applications. VLPs are uniform in size when compared with other nanoparticles. This makes them suitable for targeting and accumulating in the specific tissues [161]. Many VLPs can encapsulate siRNA, chemotherapeutic drugs, protein toxins, and other potential drugs. Ashley et al. recently reported MS2 VLPs that may encapsidate several cargos and induce selective cytotoxicity in cancer cells [162]. Thus, VLPs can be used to treat various diseases.

For VLPs to work properly in the human organism, researchers need to produce VLPs that precisely target the cells, tissues, or organs in the human body. As mentioned above, the human body has mechanisms to fight viruses. And VLPs are viral particles that are recognized by the immune system and removed from the organism. Humans are naturally exposed to certain viruses and may have antibodies that act against viruses and VLPs [64]. Therefore, the drugs produced using VLPs will not work well enough in these patients [163].

Lipid nanoparticles (LNPs) are an option to make delivery vehicles. They are widely used for mRNA vaccines. LNPs are nano-sized particulates of lipid materials. Nowadays their preferred use is to protect RNA from degradation [164]. However, LNPs may also cause side effects in the cells [165]. The main advantage of VLPs over LNPs is that VLPs can be engineered to display specific antigens or epitopes on their surface. This allows us to make customizable cargo for drug delivery.

Structural viral proteins have been used to produce VLPs. The capsid enables VLPs to enter the cell and the nucleus. Therefore, the development of well-functioning capsid proteins is crucial for the development of VLPs [64,163]. VLPs are recognized by pathogen recognition receptors (PRRs). Variations in the human genome influence the efficacy of VLP-utilizing drug formulations, such as vaccines [166]. Vaccinomics describes how genes can influence variations in the immune response and aims to develop more efficient vaccines [167,168]. In a study comprising 214 papers, genetic associations with vaccines were analyzed. Of these, six studies investigated the influence of genes on the safety of vaccines. HLA class II gene variants were most frequently associated with the immunogenicity of vaccines (such as DQB1, DRB1) [169]. Human clinical trials of HIV-1 vaccines using VLPs showed the production of antibodies against these VLPs [170]. VLPs are strong activators of dendritic cells. The activation of dendritic cells leads to the priming of immunity mediators (T and B cells). VLPs bind pattern recognition receptors on dendritic cells. Then, internalization occurs [166]. An understanding of phenotype features using genomics data may lead to the production of optimal VLPs [54] that may avoid immune system components, further deactivation, and removal. Biela and colleagues proposed a programmable polymorphism, a mechanism to control MS2 bacteriophage VLPs’ morphology by inserting amino acid sequences into an external loop. This made it possible to change the morphology to larger forms [171]. Engineering improves VLPs. The use of different glycoproteins alters the cellular tropism of VLPs. These engineered virus-like particles without genetic material are also referred to as eVLPs. The use of genome-editing tools with eVLPs for the treatment of PCSK9-dependent blindness in mice has shown good results [102].

The growing interest in VLP-based drug formulations has undoubtedly been driven by the development of molecular biology methods used to analyze the human genome more comprehensively. A deeper and more complex understanding of biological mechanisms will require more precise drug formulations and dosages in the near future. This can only be achieved through close interdisciplinary co-operation.

## 15. Conclusions

Precise therapy is better than conventional treatment methods. It can solve many problems related to drug use, like low bioavailability, frequent use, side effects, and lack of patient compliance. To address these problems, nanoparticles could be used. Viral nanoparticles have been produced that do not contain genetic material but can be utilized to envelop and transport drugs. These nanoparticles are very promising for targeting the desired receptor, cell, or tissue. However, the immune response of the host may impede the clinical application of such formulations. Human endogenous retroviruses could potentially overcome this issue. PEG10 is an auspicious candidate that can bind to mRNA and is secreted like enveloped virus-like extracellular vesicles. mRNA molecules flanked by the PEG10 gene might be encapsulated and transported to the required target. Therefore, nanoparticles derived from humans may allow us to avoid human intracellular defense mechanisms. By combining pharmacogenomics, biotechnology of nanomaterials, vaccinomics, etc., a new generation of drugs could be developed for the precise treatment of individual diseases. The concept of a specific drug for a specific patient would enable appropriate treatment of diseases that cannot be cured with the treatment methods used to date.

## Figures and Tables

**Table 1:** The main advantages and disadvantages of using expression platforms.

Platform	Advantages	Disadvantages
Bacteria	Inexpensive Robust Small protein production Chimeric VLPs	Do not have PTM Cannot form disulfide bond Cannot be used for enveloped VLPs Protein solubility problems High level of impurities
Yeast	Easy to genetically manipulate Robust Inexpensive Free of endotoxins Rapid growing Very concentrated cell density Express different forms of products Can secrete biotechnologically produced proteins into the culture medium	Protein glycosylation differs in humans and yeast Not able to produce humanized glycans Cannot produce enveloped VLPs
Mammalian	Enveloped and non-enveloped VLPs Precise PTM Large protein production Animal cells are less susceptible to human viral infection Proteins are secreted in cell culture medium	Time consuming gene introduction and protein production Contamination with fetal bovine albumin Low yield of target proteins Expensive production Infection with mammalian viruses
Plant-based	Plant-based viruses do not infect humans Rapid expression No contamination with human or animal proteins.	Sialylation of glycoprotein, durability issues

## Data Availability

Not applicable.
